# Children and Adults Prefer the Egocentric Representation to the Allocentric Representation

**DOI:** 10.3389/fpsyg.2018.01522

**Published:** 2018-08-17

**Authors:** Qingfen Hu, Ying Yang, Zhenzhen Huang, Yi Shao

**Affiliations:** ^1^Institute of Developmental Psychology, Beijing Normal University, Beijing, China; ^2^Department of Psychology, Oklahoma City University, Oklahoma City, OK, United States

**Keywords:** egocentric representation, allocentric representation, strategy preference, children, adults

## Abstract

We studied the strategy preference of using the egocentric or the allocentric representation in individuals who have acquired the ability to use both representations. Fifty-seven children aged 5–7 years and 53 adults retrieved toys hidden in one of four identical containers in a square room. We varied the type of spatial representation available in four conditions: (1) only self-motion information (egocentric representation); (2) only external landmark cues (allocentric representation); (3) both self-motion and landmark cues (dual representation); (4) self-motion and landmark cues in conflict (conflict trial). We found that, compared with the allocentric representation, the egocentric representation approached maturity earlier in development and was exploited better in early years. More importantly, in the conflict trials, while both children and adults relied more on egocentric representation, still a small portion of participants chose allocentric representation, especially in the adult group. These results provided evidence that egocentric representation is generally preferred more in both young children and adults.

## Introduction

As mobile creatures, it is crucial for us to maintain spatial information about our surrounding environment in daily life. Based on accumulating evidence, many researchers argued that there were two types of frames of reference in spatial memory: egocentric and allocentric representation (e.g., Mou et al., 2004). Egocentric representation encodes the object’s position relative to one’s body (object-to-self relation), such as “the toy is on my left"; while allocentric representation encodes a location with respect to external environment (object-to-object relation), such as “the toy is beside the desk" (e.g., Klatzky and Golledge, 1998). When moving to a new position, we have to take advantage of self-motion cues to update the egocentric representation of locations (e.g., Wang and Spelke, 2002), but this updating process will become difficult as travel distance and the number of locations remembered increase. Thus, more enduring allocentric representations may provide a better basis for flexible navigation (Burgess, 2006; Nardini et al., 2006).

A body of developmental literature has examined the developmental time courses of these spatial representations. Some early studies suggested that egocentric response to spatial stimuli predominates in early childhood (e.g., Piaget and Inhelder, 1967; Acredolo, 1978). There was evidence for egocentric spatial coding from early infancy. For example, 9-month-old infants were able to retrieve a hidden object after they were moved along simple paths of rotation or translation, which indicated they could update the egocentric spatial relations in simple movements (Landau and Spelke, 1988). Studies with young children also showed that after moving to the opposite side of the room, 4-year-olds succeeded in finding the correct place (either to their left or right) from a different view (Acredolo, 1978).

The ability to use allocentric frames in active search tasks emerges sometime in the second year. In a typical task, children went through a disorientation procedure, which prevented them from tracking changes in the egocentric relations as they move, and had to rely on external cues to retrieve the hidden object. It was found that 1.5- to 2-year-olds could use the rectangular shape of the testing room (Hermer and Spelke, 1994, 1996) as well as the left/right sense of the colored landmarks (Nardini et al., 2008) to reorient themselves. However, this ability was not stable until the age of 4 or 5, and children’s competence showed in these tasks depended on the specific type of external cues used and task details (Cheng and Newcombe, 2005; Hupbach and Nadel, 2005).

To summarize, most previous research has focused on the separate developmental trajectories of different types of spatial representations, and revealed that egocentric representation developed and reached maturity earlier than allocentric representation. To the best of our knowledge, only two studies have investigated children’s capacity to use or preference for the egocentric and the allocentric representation in location memory tasks. In Nardini et al.’s (2006) study, children aged 3–6 years retrieved a hidden toy from an array of 12 identical containers. In some conditions, either egocentric or allocentric representation was eliminated to test the effect of these reference frames. The results demonstrated the parallel operation of egocentric and allocentric frames and their additive effect on spatial memory from 3 years. Bullens et al. (2010) examined children’s route navigation performance in virtual reality environment. They found that children aged 5–10 years performed above chance when the allocentric strategy was imposed but a majority of them spontaneously used the egocentric strategy.

Yet the methods used in these two studies have some deficiencies in comparing children’s ability to use egocentric and allocentric representation directly. Nardini et al.’s (2006) work mainly focused on the additive effect of the two frames, and thus the effect of each frame was revealed by children’s better performance when both frames were available compared to when only single frame was available. However, simple egocentric or allocentric representation was not tested precisely in their paradigm. In the condition aimed at egocentric representation, the hiding place was held constant relative to the participant’s body, which did not involve updating by self-motion. Whereas in the condition aimed at allocentric representation, the original hiding place changed during the movement, but the participant could update the egocentric representation via self-motion cues. That is, egocentric reference system was still available under the circumstance. By contrast, Bullens et al.’s (2010) study focused on the development of spontaneous exploitation of these strategies. Consequently, their study did not test the egocentric strategy separately. In addition, their study asked children to navigate in a StarMaze including five alleys, which was too hard for children. Furthermore, in these two studies, the difficulty of using egocentric and using allocentric representation in the spatial task differed significantly. In the egocentric task, participants usually only undergo a slight movement and perspective change, whereas, in the allocentric task, participants generally need to encode and use complex external cues. Therefore, egocentric and allocentric representations have hardly been compared in an equivalent way.

In addition, a theoretically interesting question has not been investigated in previous research: When children have acquired the ability to use these two representations to encode locations, do they rely more on one of them when both are available? While egocentric representation receives priority in development, emerging earlier and being better used, does it also show a priority in spatial memory process when capacities of using these representations reach maturity?

Despite a lack of direct empirical evidence on these questions, we can get some hints about the answers from some theories and studies. Wang and Spelke (2000, 2002) argued that human’s navigation and location coding primarily depend on the egocentric representation. The external spatial information, such as geometry of the environment, helps individuals to reorient under the circumstances that they cannot maintain their spatial relations with the external environment. So according to this proposition, allocentric representation would not be used unless egocentric representation was not able to play its due role. That is to say, when both representations are available, human would prefer to rely on the egocentric one. By contrast, in a study to test the formation of these two representations, Pasqualotto et al. (2013) found that congenitally blind participants performed better on using egocentric reference frame, while sighted participants showed a preferential use of allocentric reference frame. These findings suggested that the development of allocentric representation requires visual input whereas the egocentric representation might be innate. Additionally, a perspective taking task has also provided evidence that children behave more egocentrically than adults and adults can correct the egocentric interpretation more successfully (Epley et al., 2004). Consequently, could we infer that egocentric representation dominates in the early years and allocentric representation gains its weight throughout the development?

**FIGURE 1:**
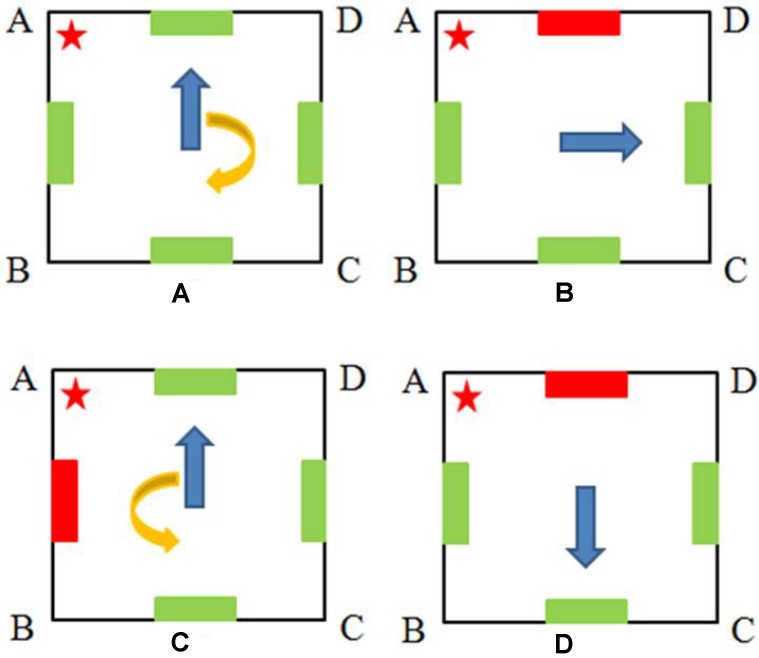
Illustration of four types of tasks in the present study: **(A)** the egocentric representation task; **(B)** the allocentric representation task; **(C)** the dual representation task and the learning phase of the conflict task; **(D)** the testing phase of the conflict task (notice that the position of the landmark changed from the AB side to the AD side during the rotation). The red star and the red bar stand for the hiding place and the distinctive landmark, respectively. The blue arrows indicate the participants’ facing directions before **(A,C)** and after **(B,D)** the rotation. The yellow arrows indicate the 180° rotations participants made. Participants were disoriented in the allocentric representation task but only went through a 180° rotation in the other three tasks. All participants went through the four tasks in the following order: dual representation-conflict-egocentric and allocentric representation (these two were counterbalanced across participants).

The present study was concerned with the developmental course of relative weighing of egocentric and allocentric representations in individuals who have acquired the ability to use both representations. We developed four conditions of hide-and-find task to examine participants’ ability to use each representation and their reliance on them. The basic task of our study was to retrieve a toy hidden in one of four identical containers in a square room. In the egocentric representation condition, there were no discriminable cues in the environment. Participants only went through a 180° rotation before retrieval (see **Figure 1A**), so they could only use the self-motion information to update the egocentric relation between their body and the target location. In the allocentric representation condition, participants were disoriented before retrieval but a distinctive landmark was provided (see **Figure 1B**), so they could only use the allocentric relation between the landmark and the target location. We controlled the spatial relation involved in both conditions. In the egocentric condition, the target location was on the left (or the right) side of the participant’s initial heading direction, so they could form an egocentric representation of “on my left.” In the allocentric task, the target location was on the left (or the right) side of the landmark, so they could form an allocentric representation of “on its left.” In the dual representation condition, there was a distinctive landmark in the environment and participants only went through a 180° rotation before retrieval (see **Figure 1C**), so they could use either spatial reference frame to encode the target location. In the conflict condition, the learning phase was the same as the dual representation condition (see **Figure 1C**). However, when the participant was going through a 180° rotation with eyes covered, we unnoticeably changed the position of the landmark. That is, the landmark was moved from the AB side to the AD side in **Figure 1D**. As a result, in the testing phase, the egocentric and the allocentric representations were in conflict, indicating two different target locations. Based on the egocentric representation updated by the self-motion information, corner A would be the appropriate choice, while based on the allocentric representation of the location (the right side of the landmark), corner D would be the correct one. Therefore, participants’ searching behaviors would provide evidence about which representation they relied on.

The present study tested children aged 5–7 years because previous research showed that the ability to reliably use both the egocentric and the allocentric representation for location coding emerged at this age (Nardini et al., 2006; Bullens et al., 2010). Their performance was compared to a group of adults, who were able to use both representations proficiently. We tested participants’ ability of using egocentric and allocentric representations separately, and further focused on those who were successful in using both representations and examined their weighing of these representations in the conflict condition. To sum up, our study investigated how the two age groups relied on these spatial representations and whether there were developmental differences between them.

Based on the previous research which indicated egocentric representation emerges earlier (Acredolo, 1978; Landau and Spelke, 1988; Cheng and Newcombe, 2005; Hupbach and Nadel, 2005), we believed children in this study will have higher accuracy in the egocentric task than in the allocentric task. But we were more enthusiastic to see their preference of these two representations. If the earlier-acquired egocentric representation also received priority in spatial memory process, at least young children would rely on it more.

## Materials and Methods

### Participants

Nineteen 5-year-olds (*M* = 59.9 months, range = 55–65 months; 10 girls), eighteen 6-year-olds (*M* = 69.4 months, range = 66–76 months; nine girls), and twenty 7-year-olds (*M* = 83.9 months, range = 78–89 months; seven girls) from Beijing, China took part in the present study. All parents gave written informed consent prior to the study. We also recruited 53 adult participants (26 women, *M* = 23 years, aged 19–35 years) from Beijing Normal University with monetary compensation and they all signed an informed consent. Six additional children were excluded from data analyses because they refused to complete the trials, and seven additional adults were excluded for guessing the purpose of this experiment.

### Apparatus

**FIGURE 2:**
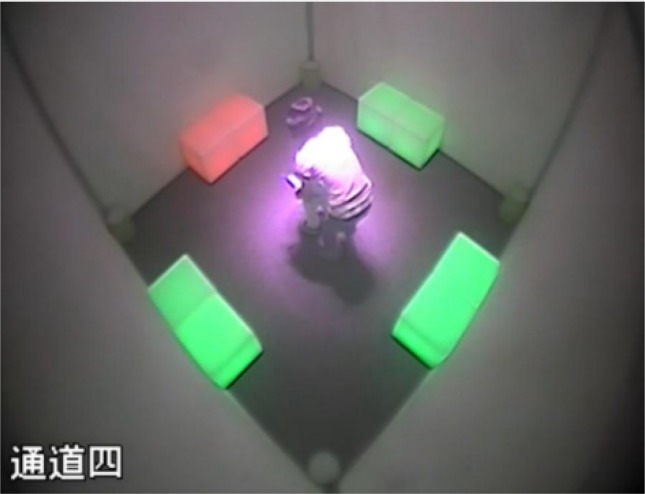
The environment and apparatus used in the present study.

Participants were tested in a square room (3 m × 3 m, 2.8 m in height). Four walls and the ceiling of the room were covered with featureless white fabric, and the floor was covered with a homogeneous gray carpet, which did not provide any visual cues. Two luminous LED cubes (80 cm × 80 cm × 80 cm) were placed in the middle of each side of the room, whose color could be changed by a remote control (see **Figure 2**). Four identical opaque inverted containers served as potential hiding places, placed in the corners of the room, and a small stuffed animal was used as the hiding object.

### Procedure

Participants were tested individually by a female experimenter. For any given participant, the hiding place was constant across trials. All participants went through four conditions. In order to obtain a precise measurement of participants’ choice in the conflict task and avoid potential bias from the egocentric or allocentric representation task, the dual representation task was always conducted first; then the conflict task was displayed, followed by the other two tasks, whose order was counterbalanced across participants. We would describe these tasks in details below.

#### Egocentric Representation Task

In this task, all cubes had identical green color. The participant first stood in the center of the room, facing a predetermined direction (see **Figure 1A**, AB or AD side), and watched while the experimenter took the toy to one corner (see **Figure 1A**, corner A) and hid it in the container. The participant was told: “You should remember where the toy is. Afterward, we will start the rotation. After the rotation, your task is to look for the toy.” After checking the participant’s memory of the hiding place, the experimenter stood behind the participant, covered his or her eyes using her hand, and spun the participant slowly until completing 180° rotation in the clockwise or the counter-clockwise direction. Finally, the participant’s eyes were uncovered and he/she was asked to find the hidden toy. Although the participants were allowed to search for the toy until they found it, only the first choice was scored for accuracy. After the toy was found, next trial proceeded.

There were four trials in this task, the order of which was counterbalanced across participants. We varied the side a participant faced before rotation (AB or AD side) and the direction a participant rotated (clockwise or counter-clockwise) in the four trials, and each combination occurred only once for each participant.

#### Allocentric Representation Task

In this task, there were red cubes on one side (AD) and green cubes on the other three sides of the room, so the red cubes could be used as landmarks (see **Figure 1B**). The experimenter first hid the toy in one container while the participant watched. The disorientation procedure then ensued as the participant faced the hiding place, his/her eyes were covered and was spun slowly by the experimenter until completing at least four full rotations in the clockwise direction and four full rotations in the counter-clockwise direction. After disorientation was confirmed, the experimenter faced the participant toward a predetermined wall. Then, the participant was asked to open his/her eyes and search for the toy, and only the first choice was scored for accuracy.

This task also consisted of four trials. For each participant, the facing directions after the disorientation procedure varied from trial to trial and the order of these trials was counterbalanced across participants.

#### Dual Representation Task

The apparatus was similar to the one used in the allocentric representation task, with distinct-color (red) cubes on one side and identical-color (green) cubes on the other three sides of the room, and the procedure was the same as the egocentric representation task (see **Figure 1C**, note that a 180° rotation would be made in this condition as in the egocentric task). There were four trials in this task, the order of which was counterbalanced across participants. We varied the side a participant faced before rotation (AB or AD side) and the direction a participant rotated (clockwise or counter-clockwise) in the four trials, and each combination occurred only once for each participant.

#### Conflict Task

The apparatus and the basic procedure were the same as the dual representation task, with the following exception. When the participant was going through the 180° rotation with eyes covered, the experimenter changed the position of the landmark (red cubes) via a remote control (before rotation, see **Figure 1C**; after rotation, see **Figure 1D**). We recorded the first corner each participant searched after the rotation. Only one trial was conducted in the conflict condition.

## Results

### When One Representation Was Available

In the egocentric and the allocentric representation condition, we compared participants’ first responses with the accurate location where the toy was hidden, and calculated accuracy scores (percent of correct responses) for each participant. The average accuracy scores of these two tasks in each age group (see **Table 1**) were all significantly above chance (25%), *p*s < 0.05 (one-sample *t*-tests).

**Table 1:** Mean accuracy scores of the egocentric and the allocentric representation condition in each age group.

		Egocentric representation condition		Allocentric representation condition	
Age group	*N*	*M*	*SD*	*M*	*SD*
5-year-olds	19	0.93	0.16	0.50	0.39
6-year-olds	18	0.88	0.21	0.58	0.30
7-year-olds	20	0.99	0.06	0.86	0.24
Adults	53	0.99	0.05	0.92	0.15

Analyses of one-way ANOVA revealed that the effect of age was significant in each condition [egocentric representation condition, *F*(3,106) = 5.08, *p* = 0.003, η_p_^2^ = 0.13; allocentric representation condition, *F*(3,106) = 18.08, *p* < 0.001, η_p_^2^ = 0.34] although Games–Howell *post hoc* comparisons revealed that the difference did not specifically occur between any of the two groups in the egocentric representation condition (*p*s > 0.05). Meanwhile, in the allocentric representation condition, the 5-year-olds performed significantly worse than the 7-year-olds (*M_D_* = 0.36, *p* = 0.008) and adults (*M_D_* = 0.42, *p* = 0.001); the 6-year-olds performed significantly worse than the 7-year-olds (*M_D_* = 0.28, *p* = 0.016) and adults (*M_D_* = 0.33, *p* = 0.001), and no other significant group difference was found.

When comparing performance level between these two conditions, paired-samples *t*-tests showed that participants searched more accurately in the egocentric representation condition in all age groups, *p*s < 0.05.

### When Both Representations Were Available

The average accuracy scores of the dual representation condition in each age group were presented in **Table 2**. One-sample *t*-tests revealed that performance of each age group was all significantly above chance (25%), *p*s < 0.001. These results indicated that all groups were able to complete the searching task in which both egocentric and allocentric representations were available.

**Table 2:** Mean accuracy scores of the dual representation condition in each age group.

		Dual representation condition	
Age group	*N*	*M*	*SD*
5-year-olds	19	0.88	0.26
6-year-olds	18	0.88	0.21
7-year-olds	20	1.00	0.00
Adults	53	1.00	0.03

### When Two Representations Were in Conflict

In order to examine the preference between two representations, although all participants completed the conflict trial, we only analyzed the searching behaviors of those who succeeded in at least three out of four trials (accuracy rate ≥75%) in both the egocentric and the allocentric conditions. Thirty-three children (*M* = 76.3 months, range = 58–89 months; six 5-year-olds, nine 6-year-olds, and eighteen 7-year-olds) and 50 adults met this criterion.

Participants’ choices in the conflict condition were distributed between corner A and corner D, so we calculated the proportions of these two choices in children and in adults, respectively (see **Table 3**). The results showed that both children and adults preferred to choose corner A, which was consistent with the egocentric representation (binomial test: children, *p* < 0.001; adults, *p* = 0.007). Nonetheless, the Mann–Whitney test revealed a marginally significant effect of age group (*Z* = -1.89, *p* = 0.059), which showed that a decreased tendency of selecting egocentric representation among adult participants.

**Table 3:** Proportions of choice of the egocentric and the allocentric representation in the conflict condition.

Participant group	Choosing corner A (egocentric representation)	Choosing corner D (allocentric representation)
Children	0.88	0.12
Adults	0.70	0.30

## Discussion

In the present study, we created two corresponding conditions, in which only one type of spatial representation (either egocentric or allocentric) could be used to relocate the hiding location. The spatial relations exploited in these tasks were similar, namely, left/right sense of self and left/right sense of a colored landmark, which enabled us to compare the results of the two conditions. We found that the 5- and 6-year-olds searched less accurately than the older age groups in the allocentric representation task, while their performance did not differ from older groups in the egocentric representation task. Additionally, all groups achieved higher accuracy in the egocentric task than in the allocentric task. These results indicated that compared with the allocentric representation, the egocentric representation approached maturity earlier in development and was exploited better especially in early years. This developmental trajectory was consistent with other previous research demonstrating an advantage for employing egocentric representations to code locations in early childhood (e.g., Bremner and Bryant, 1977; Bullens et al., 2010; Ruggiero et al., 2016), and has been suggested to be connected to the delayed maturation of hippocampus and surrounding areas (Overman et al., 1996; Newcombe and Huttenlocher, 2003).

More importantly, by incorporating both the egocentric and the allocentric representation into a novel transformational condition, we investigated which type of representation has priority for use when the ability to use these representations has matured. Only participants who succeeded in using both types of representations were included in the analysis. The results revealed that both children and adults relied on the egocentric representation more frequently than the allocentric representation, which indicated the predominant role of egocentric representation. The transformational approach has proven to be a valid measure of the relative weighing of different types of spatial cues (such as length and angular cues) in animals and human adults (e.g., Lubyk et al., 2012). In the present study, we used this approach to determine which reference frame did participants rely on, and adapted it for young children for the first time. No children and only a few adults (excluded from analysis) detected the transformation. In fact, those children and adults who searched at the corner consistent with the allocentric representation went directly to this corner and appeared surprised when they failed to retrieve the hidden toy in their first attempt. So this task is a valid test to distinguish between different strategies based on participants’ searching behaviors and provides a useful method for future studies.

Our results provide clear evidence that the egocentric reference frame, which is dominant in development, does have priority when the ability to use various spatial frames matures. What’s more, one thing to note is that indeed a small portion of participants preferred allocentric representation in both age groups. It is not likely that their performance could be attributed to random variation since they consistently chose the corner corresponded to allocentric representation, rather than the other two corners. These results are inconsistent with Wang and Spelke’s (2000, 2002) theory, and clearly demonstrate that some individuals encode, retain, and more importantly, choose to rely on allocentric representation even if the egocentric representation is still available. A further interesting question is that whether this is a stable strategy chosen only by a small part of the population or serves as a potential alternative for all individuals. Future research could take a closer look at these possibilities.

When comparing the performance of children and adults in the present task, we found that a higher proportion of adult participants selected the allocentric representation in the conflict situation. Pasqualotto et al. (2013) suggested in their study, the egocentric and allocentric representations have different origins: egocentric representation is innate, while allocentric representation derives from acquired visual experience. Based on this proposition, it is reasonable to speculate that egocentric representation, as a more primitive form of spatial representation, plays a more important role in early development. Then what developmental mechanism leads at least some individuals to shift to reliance on allocentric representation? Our findings showed that adult participants not only selected the corner consistent with allocentric representation more frequently in the conflict condition, but also achieved a higher accuracy in the allocentric condition. Does it suggest that young children’s less weighing of allocentric representation is due to their lower ability to use this type of representation? Future research could further investigate whether the transition from egocentric to allocentric representation results from a shift in preference of spatial cues in environment, or it simply implies gradual proficiency in using a variety of spatial information. A further interesting question is whether this pattern pf preference maintains stable during the development. In a study using a Y-maze task, researchers found that egocentric strategy was more preferred in old adults than in young adults (Rodgers et al., 2012). This issue could be tested using the method of this study. Another topic remained to be explored is how to interpret the individual difference in strategy preference. For instance, whether there is a causal relation between an individual’s spatial ability and his/her strategy of using different types of representations, and how this relation contributes to the development of individual difference in strategy preference. Future research may also explore the preference of children and adults to use these two representations in large and more complex environment.

## Conclusion

To conclude, although humans are able to represent locations egocentrically as well as allocentrically, they seem to prefer the exploitation of the egocentric relations. Apart from the findings consistent with previous research, the present study also provides clear evidence that allocentric representation is weighed more heavily by some individuals or in some cases, and this preference increases from early childhood to adulthood. These results raise a few valuable issues for future research about the role of egocentric and allocentric representations in spatial navigation and how they change over development. Future research may also explore the preference of children and adults to use these two representations in large and more complex environment.

## Ethics Statement

The protocol of the study was approved by the Ethics Review Committee at the School of Psychology, Beijing Normal University. For child participants, parents gave written informed consent prior to the study. Child participants gave oral assent prior to the study. Adult participants signed informed consent prior to the study.

## Author Contributions

QH developed the study concept. QH and YY created the study design. YY performed testing and data collection. QH and YY performed the data analysis and interpretation. QH, ZH, and YY drafted the manuscript. YS revised the manuscript. All authors approved the final version of the manuscript for submission.

## Conflict of Interest Statement

The authors declare that the research was conducted in the absence of any commercial or financial relationships that could be construed as a potential conflict of interest.
